# A High-Performance InGaAs Vertical Electron–Hole Bilayer Tunnel Field Effect Transistor with P^+^-Pocket and InAlAs-Block

**DOI:** 10.3390/mi14112049

**Published:** 2023-10-31

**Authors:** Hu Liu, Peifeng Li, Xiaoyu Zhou, Pengyu Wang, Yubin Li, Lei Pan, Wenting Zhang, Yao Li

**Affiliations:** School of Electronic and Information Engineering, Lanzhou Jiaotong University, Lanzhou 730070, China

**Keywords:** tunnel field effect transistor, line tunneling, P^+^-pocket, InGaAs/InAlAs

## Abstract

To give consideration to both chip density and device performance, an In_0.53_Ga_0.47_As vertical electron–hole bilayer tunnel field effect transistor (EHBTFET) with a P^+^-pocket and an In_0.52_Al_0.48_As-block (VPB-EHBTFET) is introduced and systematically studied by TCAD simulation. The introduction of the P^+^-pocket can reduce the line tunneling distance, thereby enhancing the on-state current. This can also effectively address the challenge of forming a hole inversion layer in an undoped InGaAs channel during device fabrication. Moreover, the point tunneling can be significantly suppressed by the In_0.52_Al_0.48_As-block, resulting in a substantial decrease in the off-state current. By optimizing the width and doping concentration of the P^+^-pocket as well as the length and width of the In_0.52_Al_0.48_As-block, VPB-EHBTFET can obtain an off-state current of 1.83 × 10^−19^ A/μm, on-state current of 1.04 × 10^−4^ A/μm, and an average subthreshold swing of 5.5 mV/dec. Compared with traditional InGaAs vertical EHBTFET, the proposed VPB-EHBTFET has a three orders of magnitude decrease in the off-state current, about six times increase in the on-state current, 81.8% reduction in the average subthreshold swing, and stronger inhibitory ability on the drain-induced barrier-lowering effect (7.5 mV/V); these benefits enhance the practical application of EHBTFETs.

## 1. Introduction

Due to the emergence of the cloud, big data and real-time data transmission have become the main trends in the development of information technology, and they require integrated circuits to have ultra-low power dissipation. However, as the core of current integrated circuits, MOSFETs suffer from the increasing static power consumption with the decrease in feature size, inhibiting the development of integrated circuits. The decrease of subthreshold swing (*SS*) is an effective approach to deal with this issue. Limited by the injection mechanism of thermal emission, the *SS* of MOSFETs cannot be lower than 60 mV/dec; thus, there is an urgent need to develop steep *SS* (<60 mV/dec) devices to satisfy the cloud applications.

The tunnel field effect transistor (TFET) [1,2,3], as a steep *SS* device, has received widespread attention because of its CMOS process compatibility and low standby power consumption. However, the on-state current (*I*_on_) of TFETs is too low for reasonable performance, which makes most research focus on overcoming this drawback. Therefore, TFETs with new structures or operation mechanisms have appeared in large numbers, such as two-dimensional material TFETs [4,5,6], negative capacitance TFETs [7,8,9], heterojunction TFETs [10,11,12], nanowire TFETs [13,14,15], line tunneling (L-tunneling) TFETs [16,17,18,19,20,21,22,23,24,25,26,27,28,29], etc. Comprehensive analysis shows that expanding the tunneling area based on the L-tunneling mechanism is a very effective approach to improving *I*_on_. The electron–hole bilayer TFET (EHBTFET) [19,20,21,22,23,24,25,26,27,28,29] is a new type of L-tunneling TFET that was first proposed by Lattanzio [19] and has been developed in recent years because of its novel tunneling mechanism. Different from the L-tunneling TFETs with the L/U/T-type gate structure [16,17,18] that create the L-tunneling by overlapping the gate and heavily doped source region, EHBTFETs can generate the L-tunneling perpendicular to the channel in the electron–hole bilayer formed by the gate engineering or bias-induced method. So far, research has mainly focused on the transverse EHBTFETs, from which it has been found that the vertical L-tunneling not only boosts *I*_on_ but also leads to high off-state current (*I*_off_). To solve this issue, many methods, such as counterdoping in the gate underlap region [23], partial light doping in the source and drain regions [24], heterogate structure [25], and implantation of a dielectric barrier layer in the gate underlap region [26] have been adopted, and *I*_off_ has been suppressed to a certain extent. However, it should be noted that the *I*_on_ of the transverse EHBTFETs is directly proportional to the gate overlap area, which means that the increase of *I*_on_ is at the expense of chip density. To simultaneously improve device performance and chip density, new EHBTFETs need to be investigated.

Vertical EHBTFETs [27,28,29] can improve *I*_on_ by expanding the tunneling area in the vertical direction without sacrificing the chip density, which attracts researchers’ attention. To further improve *I*_on_, III-V materials are usually adopted in vertical EHBTFETs due to their smaller electron effective mass and band gap (*E*_g_) [30], but which can also exacerbate the deterioration of *I*_off_. Our previous research [29] demonstrates that the impact of the point tunneling (P-tunneling) between the gate underlap region and the drain on the *I*_off_ is significant, which cannot be effectively attenuated through the approaches used in transverse EHBTFETs. Although our proposed DGNP-EHBTFET in Reference [29] can solve this problem well, *I*_on_ can only be maintained without degradation and cannot be improved. To obtain better off- and on-state device performance, an improved vertical EHBTFET is proposed in this paper, namely, an In_0.53_Ga_0.47_As EHBTFET with a P^+^-pocket and an In_0.52_Al_0.48_As-block (VPB-EHBTFET). Since the line tunneling of EHBTFETs depends on the concentration of the electron–hole bilayer in the channel, the P^+^-pocket can be used to generate the hole layer in real operation. This is due to that for high-K/InGaAs interface, a large number of interface states near the valence band in InGaAs will inhibit the formation of the hole layer in the InGaAs channel without any acceptor doping. Although some methods, such as the use of Al_2_O_3_/HfO_2_ bilayer gate oxide [31], the postdeposition annealing process [32], and the gate-last process [33], can effectively reduce the interface trap density for the HfO_2_/InGaAs interface, P-type doping performed in the right-side channel may be the best method for creating the hole layer in real operation. More importantly, the P^+^-pocket can reduce the tunneling distance of the L-tunneling, which enhances the electron tunneling and achieves the goal of improving the on-state current. In_0.52_Al_0.48_As possesses greater *E*_g_ and carrier effective mass and matches the lattice of In_0.53_Ga_0.47_As. Due to its excellent material properties, placing In_0.52_Al_0.48_As in the gate underlap region near the drain can inhibit the point tunneling in this region. Moreover, it can also effectively avoid potential performance degradation caused by the lattice mismatch in the device fabrication. Heretofore, since investigations on suppressing off-state leakage and improving on-state performance for vertical EHBTFETs are relatively few, revealing the physical mechanism of the proposed VPB-EHBTFET is necessary, thereby providing theoretical guidance for device manufacturing. 

The content of this paper focuses on the following aspects. Device structures and corresponding parameters, key manufacturing processes, and physical models for simulations are introduced in Section 2. Section 3 examines in detail the performance comparison between conventional and improved EHBTFETs and the effects of P^+^-pocket and InAlAs-block on the proposed VPB-EHBTFET. Finally, a concise summary of the current studies is provided in Section 4.

## 2. Device Structures and Simulation Methods

**Figure 1:**
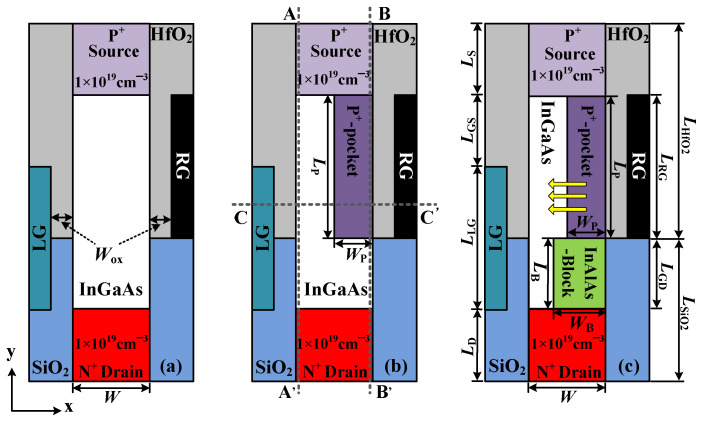
Schematics of (**a**) V-EHBTFET; (**b**) VP-EHBTFET; and (**c**) VPB-EHBTFET.

For comparison, we bring in two other EHBTFETs, namely, (1) a traditional vertical EHBTFET (V-EHBTFET) and (2) a vertical EHBTFET with a P^+^-pocket (VP-EHBTFET). Cross-sectional views and corresponding device parameters of the three EHBTFETs are given in Figure 1 and Table 1, respectively. It is found that EHBTFETs’ difference only exists in the channel. The bulk material of the three EHBTFETs is In_0.53_Ga_0.47_As, but there is an In_0.52_Al_0.48_As-block in the channel of VPB-EHBTFET compared with the other two devices. Moreover, only the channel near the right gate (RG) of VP-EHBTFET and VPB-EHBTFET possesses the P^+^-pocket with the doping concentration of 6 × 10^19^ cm^−3^. For these three EHBTFETs, since the L-tunneling occurs in the electron–hole bilayer of the gate overlap region (GO region) (see yellow arrows in Figure 1c), the appropriate carrier concentration is required in this region. According to the charge plasma concept [34], chromium (work-function = 4.5 eV) is employed as the left gate (LG) to induce a two-dimensional electron gas layer, and aurum (work-function = 5.3 eV) is adopted as RG to create a two-dimensional hole gas layer [35]. To induce a uniform carrier distribution, the width of bulk material is set to 10 nm. Furthermore, some key material parameters used in simulations refer to reference [10].

The proposed VPB-EHBTFET can be fabricated with the most advanced process technology currently available, in which the In_0.53_Ga_0.47_As epitaxial layer with the In_0.52_Al_0.48_As-block can be grown vertically on the InP substrate by molecular beam epitaxy technology, and the P^+^-pocket is created by ion implantation. Inductively coupled plasma etching is employed to etch dielectric and epitaxial layer materials, and the atom layer deposition technique can be adopted to deposit dielectrics and metal electrodes. The key process is the fabrication of the P^+^-pocket and the In_0.52_Al_0.48_As-block, which is closely related to the accurate control of doping depth and concentration in the ion implantation, as well as the precise design of the mask pattern.

All device simulations are performed using the Silvaco-Atlas 2-D numerical simulation platform. In simulations, the density gradient model is included to take into account the quantum confinement effect. Additionally, models included in references [10,29] are considered, such as the non-local band-to-band tunneling (BTBT) model, the Lombardi mobility model, the strained two-band zincblende model, etc. To compare under the same conditions, the influence of trap is not considered in the simulations. 

## 3. Results and Discussion

### 3.1. Performance Comparison between V-EHBTFET, VP-EHBTFET, and VPB-EHBTFET

Referring to our previous research on EHBTFETs [29], it is known that there are two tunneling mechanisms in this kind of device, namely, the P-tunneling and L-tunneling parallel to and perpendicular to the channel, respectively. The P-tunneling basically occurs in the gate underlap region near the source or drain (named GUS region or GUD region, respectively), while the L-tunneling occurs in the GO region. Both tunneling mechanisms are closely related to *P*_tun_, which can be expressed as Equation (1) [29]:

(1) $$P_{\mathrm{tun}}\propto \exp\left(-\frac{4\lambda \sqrt{2m*}E_{g}^{3/2}}{3\;|\;e\;|\;\hbar (E_{g}+\Delta \varphi )}\right)=\exp\left(-\frac{4\sqrt{2m*}E_{g}^{3/2}}{3\;|\;e\;|\;\hbar E}\right)$$

**Figure 2:**
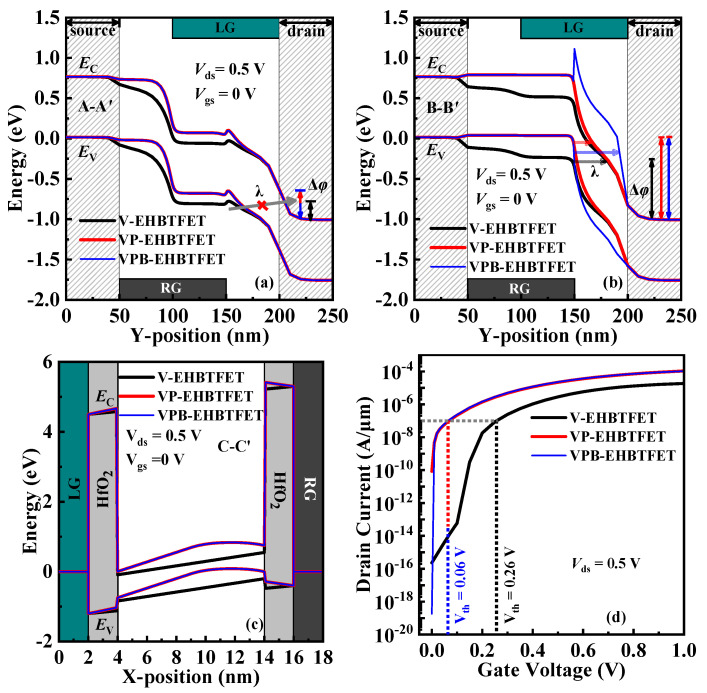
Off-state energy band profiles for EHBTFETs along (**a**) A-A′, (**b**) B-B′, and (**c**) C-C′, respectively; (**d**) *I*_ds_-*V*_gs_ curves of EHBTFETs.

Two key factors affecting *P*_tun_, the tunneling distance (*λ*) and the energy range used for tunneling (Δ*φ*), can be extracted from the energy band. Therefore, to interpret the tunneling mechanism in detail, energy bands in the off-state [gate voltage (*V*_gs_) = 0 V, drain voltage (*V*_ds_) = 0.5 V] for these three EHBTFETs are calculated along A-A′, B-B′, and C-C′ (gray dotted lines in Figure 1b), respectively, and plotted in Figure 2a–c, respectively.

As shown in Figure 2a, although Δ*φ* appears in the GUD region, the P-tunneling in the left-side channel of the three EHBTFETs is suppressed due to the very long *λ*. It is observed from Figure 2b that the P-tunneling in the right-side channel also occurs in the GUD region. Since the introduction of a P^+^-pocket can enlarge Δ*φ* and decrease *λ*, the P-tunneling in the off-state for VP-EHBTFET is stronger than that for V-EHBTFET, according to Equation (1). Although VPB-EHBTFET also possesses the P^+^-pocket like VP-EHBTFET, its P-tunneling can be better suppressed compared with the other two EHBTFETs. This is because there is an In_0.52_Al_0.48_As-block with wider *E*_g_ in the GUD region of VPB-EHBTFET, which significantly degrades the *P*_tun_. Figure 2c shows the energy band profiles in the GO region, from which it is found that the L-tunneling does not take place in the off-state due to no Δ*φ*. Based on the analyses in Figure 2a–c, it is concluded that in the off-state, the P-tunneling in the right-side channel is dominant, and the proposed VPB-EHBTFET has the weakest tunneling capacity. *I*_off_ is an important parameter to test the off-state performance of devices, which can be extracted from the transfer characteristic curve (i.e., *I*_ds_-*V*_gs_ curve). To verify the above mechanism analysis, *I*_ds_-*V*_gs_ curves of these three EHBTFETs are computed and shown in Figure 2d. As observed from the figure, it results that *I*_off_ of VPB-EHBTFET approaches as low as 1.83 × 10^−19^ A/μm. Compared with the *I*_off_ of V-EHBTFET and VP-EHBTFET (2.29 × 10^−16^ A/μm and 8.37 × 10^−11^ A/μm, respectively), that of VPB-EHBTFET is reduced by approximately three and eight orders of magnitude, respectively. It follows that VPB-EHBTFET has the best off-state performance, which is consistent with the tunneling mechanism analyzed above. 

**Figure 3:**
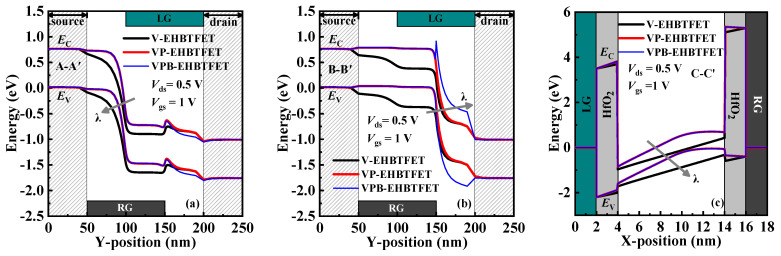
On-state energy bands for EHBTFETs along (**a**) A-A′, (**b**) B-B′, and (**c**) C-C′, respectively.

*I*_on_, a key performance indicator in the on-state (*V*_gs_ = 1 V and *V*_ds_ = 0.5 V in simulations), can also be extracted from Figure 2d. *I*_on_ of VPB-EHBTFET approaches 1.04 × 10^−4^ A/μm, which is basically the same as that of VP-EHBTFET but about 5.7 times higher than that of V-EHBTFET (1.84 × 10^−5^ A/μm). Here, we still explain their differences from the perspective of the energy band. Similarly, energy band profiles in the on-state for the three EHBTFETs are extracted and plotted in Figure 3.

From Figure 3a,b, the P-tunneling in the left-side channel occurs in the GUS region, which is different from that in the off-state, but that in the right-side channel still occurs in the GUD region. Based on the previous analysis, it can be known that the effect of the P^+^-pocket and the In_0.52_Al_0.48_As-block on the P-tunneling in the on-state is the same as that in the off-state. Combined with the P-tunneling in the left- and right-side channels, it can be inferred that the adoption of the P^+^-pocket is conducive to the enhancement of P-tunneling in the on-state. Furthermore, it is observed from Figure 3c that the energy bands in the GO region of VP-EHBTFET and VPB-EHBTFET bend upward due to the existence of the P^+^-pocket, which can reduce the *λ* of the L-tunneling and eventually enhance their L-tunneling. Since the L-tunneling occurs in the whole GO region (50 nm in length), while the P-tunneling region only exists within a range of a few nanometers near the gate, and the *λ* of L-tunneling is much smaller than that of P-tunneling, both of these cause the L-tunneling to dominate in the on-state. As a result, the tunneling ability of VP-EHBTFET and VPB-EHBTFET is basically the same in the on-state and stronger than that of V-EHBTFET, eventually resulting in better on-state performance in VP-EHBTFET and VPB-EHBTFET.

**Figure 4:**
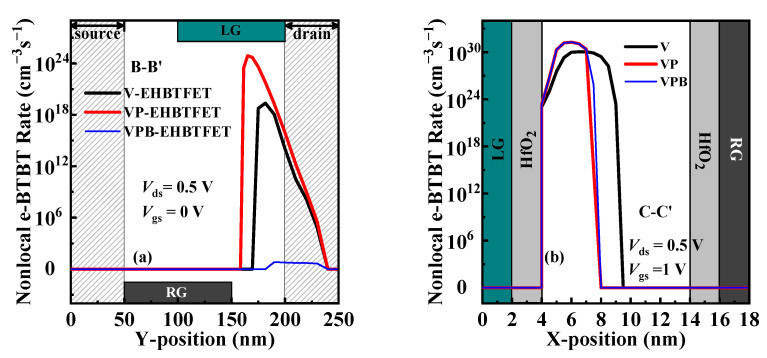
Nonlocal e-BTBT rates for EHBTFETs along (**a**) B-B′ in the off-state and (**b**) C-C′ in the on-state.

To better understand the tunneling mechanism of devices, it can be further investigated from the point of view of the non-local electron BTBT (e-BTBT) rate. According to the results of energy band analysis, only the off-state e-BTBT rate in the right-side channel and the on-state one in the GO region of V-EHBTFET, VP-EHBTFET, and VPB-EHBTFET are extracted and displayed in Figure 4a,b, respectively. It is found from Figure 4a that the peak values of the off-state e-BTBT rate for these three EHBTFETs occur in the GUD region and show the following trend: VP-EHBTFET >> V-EHBTFET >> VPB-EHBTFET, which directly reflects that the enhancement of the off-state P-tunneling by the P^+^-pocket can be suppressed significantly by the In_0.52_Al_0.48_As-block. The smaller the off-state e-BTBT rate of VPB-EHBTFET, the better its off-state performance. Figure 4b shows that the peak value of the on-state e-BTBT rate in the GO region of VP-EHBTFET and VPB-EHBTFET is the same, but it is one order of magnitude higher than that of V-EHBTFET. Thus, it can be confirmed again that the P^+^-pocket benefits improve the on-state L-tunneling, which makes the proposed VPB-EHBTFET have good on-state performance as VP-EHBTFET.

**Figure 5:**
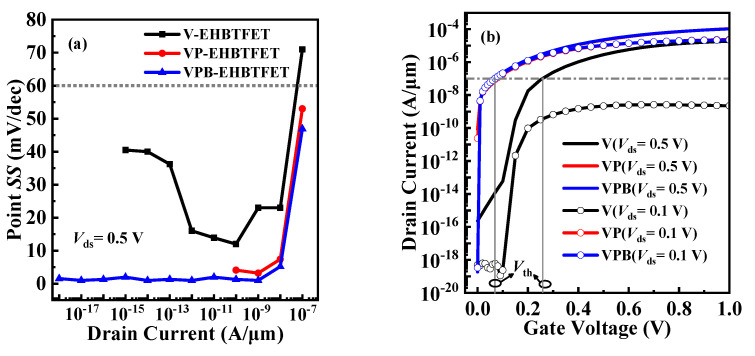
(**a**) The correlation between point *SS* and drain current of EHBTFETs; and (**b**) *I*_ds_-*V*_gs_ curves of EHBTFETs at *V*_ds_ = 0.1 V and *V*_ds_ = 0.5 V, respectively.

Furthermore, other important performance parameters, such as *I*_on_/*I*_off_, subthreshold voltage (*V*_th_), average subthreshold swing (*SS*_avg_), point subthreshold swing (point *SS*), and drain-induced barrier lowering (DIBL), are calculated based on the *I*_ds_-*V*_gs_ curves. Due to the high *I*_on_ and the minimum *I*_off_ in the proposed VPB-EHBTFET, it obtains the maximum *I*_on_/*I*_off_ of 5.7 × 10^14^. *V*_th_ usually refers to the *V*_gs_ corresponding to the midpoint of the transition zone, where the drain current (*I*_ds_) changes sharply with the *V*_gs_ in the *I*_ds_-*V*_gs_ curve. Referring to previous publications [2,10], *V*_gs_ corresponding to *I*_ds_ = 1 × 10^−7^ A/μm is taken as *V*_th_ in this paper. As shown in Figure 2d, the *V*_th_ of the proposed VPB-EHBTFET is as low as 0.06 V, which is the same as that of VP-EHBTFET. Moreover, the *V*_th_s of VPB-EHBTFET and VP-EHBTFET are less than that of V-EHBTFET (0.26 V). This is due to the introduction of P^+^-pocket reducing the *λ* of the L-tunneling, allowing VP- and VPB-EHBTFETs to be turned on at lower *V*_gs_. *SS*_avg_ is expressed as *SS*_avg_ = (*V*_th_ – *V*_off_)/(log *I*_Vth_ – log *I*_Voff_), where *V*_off_ is the *V*_gs_ at which the *I*_ds_ begins to increase. In view of the minimum *I*_off_ (i.e., *I*_Voff_) caused by the In_0.52_Al_0.48_As-block, *SS*_avg_ of 5.5 mV/dec can be obtained in VPB-EHBTFET, which is reduced by 81.8% and 75.1% compared with that in V-EHBTFET and VP-EHBTFET (30.2 mV/dec and 22.1 mV/dec, respectively), respectively. Figure 5a shows the point *SS* values of the three EHBTFETs, where the point *SS* is calculated by d*V*_gs_/d(log*I*_ds_). It is found that VPB-EHBTFET possesses steeper point *SS* at each *I*_ds_; in particular, when *I*_ds_ < 10^−8^ A/μm, the point *SS* is around 1 mV/dec and basically remains unchanged, guaranteeing the steepest *SS*_avg_ in VPB-EHBTFET. DIBL can be used to characterize the shift of *V*_th_ in devices, which is usually defined as Δ*V*_th_/Δ*V*_ds_. To obtain the DIBL values of the three EHBTFETs, the *I*_ds_-*V*_gs_ curves are calculated at *V*_ds_ = 0.1 V and 0.5 V, respectively, and plotted in Figure 5b. Since *V*_th_s of the proposed VPB-EHBTFET and VP-EHBTFET change negligibly under different *V*_ds_s, a low DIBL value of 7.5 mV/V can be achieved in both EHBTFETs. This is because the existence of the P^+^-pocket enhances the built-in electric field in the GO region, thereby reducing the influence of the applied electric field on the L-tunneling. However, the DIBL value cannot be calculated for V-EHBTFET because this device is still turned off at *V*_ds_ = 0.1 V. Thus, it can be seen that the adoption of the P^+^-pocket can effectively suppress the DIBL effect. For a clear comparison, the parameters discussed above for the three EHBTFETs are summarized in Table 2.

### 3.2. Effect of P^+^-Pocket on VPB-EHBTFET

**Figure 6:**
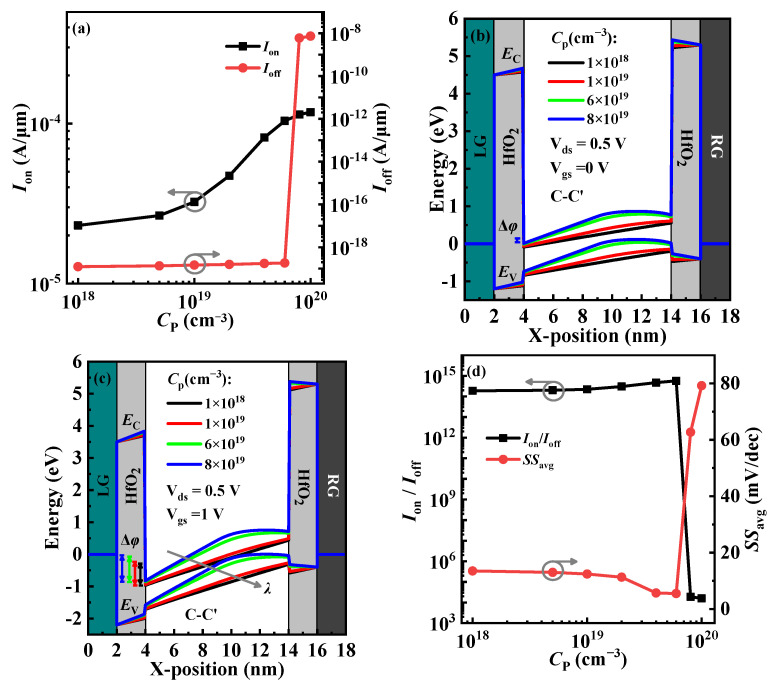
(**a**) The variation of *I*_on_ and *I*_off_ with *C*_P_ for VPB-EHBTFET; (**b**) off-state and (**c**) on-state energy band profiles along C-C′ for VPB-EHBTFET; and (**d**) the variation of *I*_on_/*I*_off_ and *SS*_avg_ with *C*_P_ for VPB-EHBTFET.

Here, doping concentration and doping width (*C*_P_ and *W*_P_) in the P^+^-pocket of the proposed VPB-EHBTFET are investigated for the optimization of device performance. Figure 6a shows *I*_on_ and *I*_off_ values under different *C*_P_s. It is found from the figure that *I*_off_ is very low and remains the same order of magnitude when *C*_P_ < 8 × 10^19^ cm^−3^, but it increases sharply with the further increase in *C*_P_. *I*_on_ increases with *C*_P_. Both of these trends can be explained by the energy band profiles. Based on the conclusion of device performance comparison between conventional and improved EHBTFETs, it is known that their *I*_off_ and *I*_on_ depend on the off-state P-tunneling in the GUD region of the right-side channel and the on-state L-tunneling in the GO region, respectively. Therefore, the off-state energy bands of the P-tunneling in the right-side channel under different *C*_P_s are calculated first, but results demonstrate that the effect of the change of *C*_P_ on the P-tunneling is negligible. This is because the existence of the In_0.52_Al_0.48_As-block makes the *λ* of the P-tunneling possess basically identical lengths (about 50 nm) under different *C*_P_s, thereby preventing the tunneling of electrons in this region. Then, the off-state energy bands of the L-tunneling in the GO region under different *C*_P_s are also calculated and plotted in Figure 6b. With the increase in *C*_P_, the energy band gradually bends upward, and a Δ*φ* can be generated when *C*_P_ > 6 × 10^19^ cm^−3^. The existence of Δ*φ* provides a condition for the off-state L-tunneling, which results in a rapid increase in *I*_off_. Moreover, for the interpretation of the change trend of *I*_on_, the on-state energy bands of the L-tunneling in the GO region are examined and shown in Figure 6c. With the increase in *C*_P_, *λ* decreases and Δ*φ* increases, so that *I*_on_ takes on a monotonically increasing trend according to Equation (1). Figure 6d shows *SS*_avg_ and *I*_on_/*I*_off_ in VPB-EHBTFET with different *C*_P_s. With the increase in *C*_P_, *SS*_avg_ decreases slowly first and then increases rapidly, but *I*_on_/*I*_off_ has the opposite change trend compared with *SS*_avg_. When *C*_P_ = 6 × 10^19^ cm^−3^, *SS*_avg_ and *I*_on_/*I*_off_ can approach the minimum and maximum values, respectively. By compromising performance parameters, it results that 6 × 10^19^ cm^−3^ is the optimal *C*_P_.

**Figure 7:**
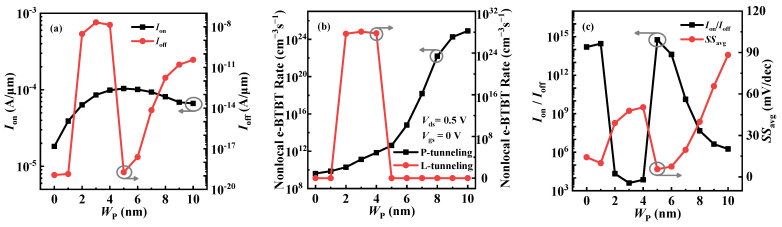
(**a**) The variation of *I*_on_ and *I*_off_ with *W*_P_ for VPB-EHBTFET; (**b**) the peak value of the nonlocal e-BTBT rate under different *W*_P_s for the P-tunneling in the left-side channel and the L-tunneling in the GO region, in the off-state; and (**c**) the variation of *I*_on_/*I*_off_ and *SS*_avg_ with *W*_P_ for VPB-EHBTFET.

Further, the effect of doping width *W*_P_ on the device performance is investigated. Figure 7a shows *I*_off_ and *I*_on_ values under different *W*_P_s. With the increase in *W*_P_, *I*_off_ increases first, then decreases, and finally increases again. To interpret this trend, we extract the e-BTBT rates that can reflect the tunneling ability of the P-tunneling and L-tunneling in the off-state and plot them in Figure 7b. It is observed from the figure that the off-state e-BTBT rates of the P-tunneling and L-tunneling are very low when *W*_P_ ≤ 1 nm, which indicates that both kinds of tunneling are suppressed in this condition, so a very small *I*_off_ can be obtained. When 1 nm < *W*_P_ < 5 nm, the off-state L-tunneling is turned on and dominates, which makes *I*_off_ have a sharp increase. This is due to the fact that the *λ* of the L-tunneling reduces with the increase in *W*_P_. With the further increase in *W*_P_, the decrease in electrons in the left side of the GO region lifts the energy band in this region; thus, the Δ*φ* of the L-tunneling starts to reduce at *W*_P_ = 4 nm and disappears at *W*_P_ = 5 nm, thereby resulting in a decrease in *I*_off_. When *W*_P_ ≥ 5 nm, the L-tunneling is turned off, and the P-tunneling is dominant, leading to *I*_off_ increasing with *W*_P_ again. This is because, with the increase in *W*_P_, the P-tunneling junction in the left-side channel becomes steeper, which increases the e-BTBT rate of the P-tunneling. *I*_on_ depends on the on-state L-tunneling in the GO region. Due to the mutual constraint between *λ* and Δ*φ*, the e-BTBT rate of the L-tunneling exhibits a trend of increasing first and then decreasing with the increase in *W*_P_, which results in the same change trend in *I*_on_ (see Figure 7a). As shown in Figure 7c, the optimal *SS*_avg_ and *I*_on_/*I*_off_ can be obtained at *W*_P_ = 5 nm. Comprehensive analysis shows that the optimal *W*_P_ is 5 nm.

### 3.3. Effect of InAlAs-Block on VPB-EHBTFET

**Figure 8:**
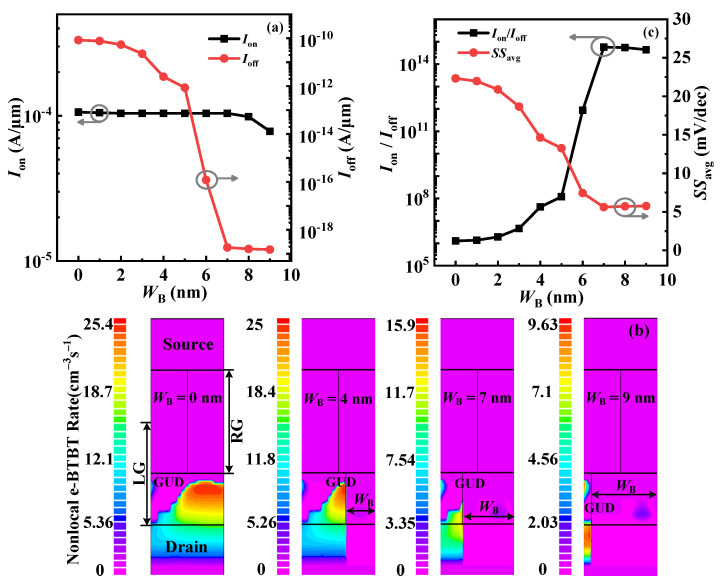
(**a**) The variation of *I*_on_ and *I*_off_ with *W*_B_ for VPB-EHBTFET; (**b**) the contour plots of the nonlocal e-BTBT rate under different *W*_B_s for VPB-EHBTFET in the off-state; and (**c**) the variation of *I*_on_/*I*_off_ and SS_avg_ with *W*_B_ for VPB-EHBTFET.

Here, the width and length (*W*_B_ and *L*_B_) of the In_0.52_Al_0.48_As-block in the proposed VPB-EHBTFET are examined to optimize device performance. Figure 8a shows *I*_off_ and *I*_on_ values under different *W*_B_s. *I*_off_ gradually decreases with the increase in *W*_B_ but basically maintains stability when *W*_B_ > 7 nm, which can be explained by the contour plots of the non-local e-BTBT rate shown in Figure 8b. It is found that both the distribution range and magnitude of the non-local e-BTBT rate reduce with the increase in *W*_B_, which can demonstrate that the increasing *W*_B_ helps to inhibit the P-tunneling in the right side of the GUD region caused by the P^+^-pocket, thereby lowering the *I*_off_. When *W*_B_ = 7 nm, the non-local e-BTBT rate falls sharply due to complete suppression of this type of P-tunneling, thus resulting in several orders of magnitude reduction in *I*_off_. As *W*_B_ goes beyond 7 nm, the In_0.52_Al_0.48_As-block begins to suppress the P-tunneling in the left side of the GUD region so as to further reduce the non-local e-BTBT rate. Since the *λ* of the P-tunneling in the left-side channel is very long, the electron tunneling in this region is insignificant. As a result, *I*_off_ is not sensitive to the change in *W*_B_. Moreover, Figure 8a shows that only when *W*_B_ > 7 nm does *I*_on_ begin to decrease. This is because although the In_0.52_Al_0.48_As-block does not affect the L-tunneling in the GO region, when *W*_B_ > 7 nm, it prevents the tunneling electrons in the left-side channel from drifting to the drain region. As shown in Figure 8c, with the increase in *W*_B_, *SS*_avg_ and *I*_on_/*I*_off_ decreases and increases, respectively, but both take on the opposite trend when *W*_B_ > 7 nm. Thus, the best choice of *W*_B_ is 7 nm.

**Figure 9:**
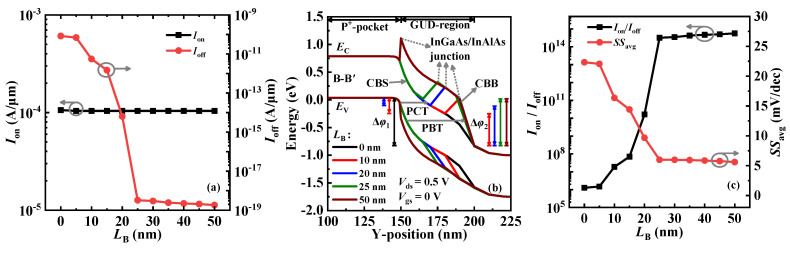
(**a**) The variation of *I*_on_ and *I*_off_ with *L*_B_ for VPB-EHBTFET; (**b**) energy band profiles of VPB-EHBTFET under different *L*_B_s in the off-state; and (**c**) the variation of *I*_on_/*I*_off_ and *SS*_avg_ with *L*_B_ for VPB-EHBTFET.

Next, the influence of *L*_B_ on the device performance is investigated. It should be noted that all research results are obtained with the unchanged channel length. Figure 9a shows the *I*_off_ and *I*_on_ values under different *L*_B_s. *I*_off_ decreases first with the increase in *L*_B_ but basically keeps stable when *L*_B_ > 25 nm. Since the off-state P-tunneling in the right-side channel is affected by the In_0.52_Al_0.48_As-block, its energy band profiles can be calculated to interpret the trend of *I*_off_. It is observed from Figure 9b that there are two kinds of P-tunneling between the P^+^-pocket and the GUD region: (1) electrons tunnel from the valence band of the P^+^-pocket into the conduction band of In_0.53_Ga_0.47_As (CBS) (named PCT-tunneling), and (2) electrons tunnel from the valence band of the P^+^-pocket into the conduction band of In_0.52_Al_0.48_As (CBB) (named PBT-tunneling). Due to the wider *E*_g_ in the In_0.52_Al_0.48_As and the longer *λ* in the tunneling junction, the effect of PBT-tunneling on *I*_off_ is negligible. When *L*_B_ = 0 nm, only the PCT-tunneling exists in the tunneling junction, and its Δ*φ* is the widest; thus, the maximum *I*_off_ is generated. With the increase in *L*_B_, the Δ*φ* of the PCT-tunneling and the PBT-tunneling (i.e., Δ*φ*_1_ and Δ*φ*_2_ in Figure 9b) decreases and increases, respectively, and disappears and saturates, respectively, when *L*_B_ ≥ 25 nm. It follows that the PCT-tunneling is gradually suppressed with the increase in *L*_B_ and eventually completely replaced by the PCB-tunneling when *L*_B_ ≥ 25 nm, thereby resulting in the change trend of *I*_off_ shown in Figure 9a. Furthermore, Figure 9a shows that *I*_on_ is immune to the change in *L*_B_, which is because the L-tunneling dominant in the on-state is not affected by the *L*_B_. Figure 9c shows that *SS*_avg_ and *I*_on_/*I*_off_ have the same and opposite change trend as *I*_off_, respectively. This is because both parameters mainly depend on *I*_off_, which is closely related to *L*_B_. This research demonstrates that good device performance can be achieved when *L*_B_ is not less than 25 nm.

**Figure 10:**
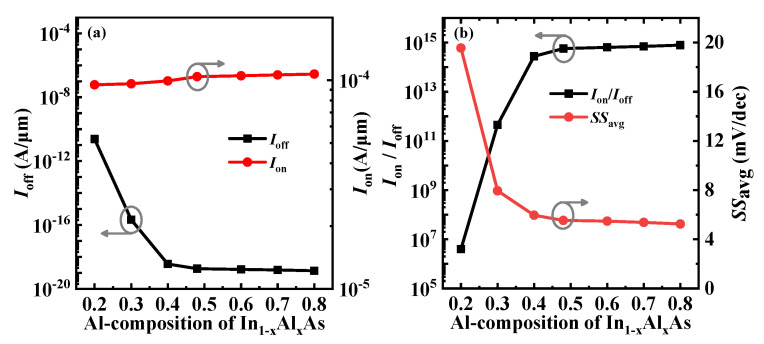
(**a**) *I*_on_ and *I*_off_; (**b**) *I*_on_/*I*_off_ and *SS*_avg_, for VPB-EHBTFET under different Al compositions.

To verify the superiority of the introduction of the In_0.52_Al_0.48_As-block, the device performance of the proposed VPB-EHBTFET under lattice mismatch was investigated, and corresponding simulation results are shown in Figure 10. In simulations, the composition of InGaAs is unchanged, while the Al composition (i.e., x) of the In_1−x_Al_x_As-block changes from 0.2 to 0.8. Considering the strain caused by the lattice mismatch, a strained two-band zincblende model is included in simulations. As shown in Figure 10a, it is observed that the *I*_on_ is insensitive to x, but the *I*_off_ decreases first with the increase in x and then basically remains unchanged when x ≥ 0.48, both of which can be interpreted by the tunneling mechanism. Based on the previous analyses, the In_1−x_Al_x_As-block located in the right-side GUD region mainly controls the off-state P-tunneling. With the increase in x, the *E*_g_ of In_1−x_Al_x_As increases. According to Equation (1), the increase in *E*_g_ benefits to reducing the *P*_tun_, eventually reducing the *I*_off_. When x ≥ 0.48, electrons are difficult to tunnel into the drain across the In_1−x_Al_x_As-block due to the long *λ* caused by the *E*_g_, which makes the *I*_off_ basically remain stable. It is found from Figure 10b that when x < 0.48, *I*_on_/*I*_off_ and *SS*_avg_ increases and decreases with the increase in x, respectively. As x increases further, both parameters tend to be saturated. Comprehensive analysis shows that good device performance of VPB-EHBTFET can be achieved when x ≥ 0.48. However, the epitaxial layers with mismatched lattices are prone to defects during growth, which can affect the performance and lifespan of devices. Therefore, In_0.52_Al_0.48_As with x = 0.48 is the optimal choice because it matches the lattice of In_0.53_Ga_0.47_As.

## 4. Conclusions

To sum up, an In_0.53_Ga_0.47_As vertical EHBTFET with a P^+^-pocket and an In_0.52_Al_0.48_As-block (VPB-EHBTFET) is introduced and investigated by TCAD simulation. Numerical simulations indicate that the adoption of a P^+^-pocket and In_0.52_Al_0.48_As-block can make VPB-EHBTFET simultaneously possess good on-state and off-state performance. Corresponding indicator parameters are *I*_on_ of 1.04 × 10^−4^ A/μm, *I*_off_ of 1.83 × 10^−19^ A/μm, *I*_on_/*I*_off_ of 5.7 × 10^14^, *SS*_avg_ of 5.5 mV/dec, and a DIBL value of 7.5 mV/V. Through examining the influence of the P^+^-pocket on VPB-EHBTFET, it results that *C*_P_ controls the device performance by affecting the L-tunneling, while *W*_P_ can simultaneously regulate the *λ* and Δ*φ* of the point tunneling and line tunneling to obtain the optimal on-state performance. Considering the changes in *W*_B_ and *L*_B_, it is concluded that both mainly control *I*_off_ through the suppression of the P-tunneling in the right-side channel. Moreover, through investigating the effect of the Al composition of the In_1−x_Al_x_As-block on the device performance and considering the potential performance degradation caused by lattice mismatch during device manufacturing, the In_0.52_Al_0.48_As-block that matches the lattice of In_0.53_Ga_0.47_As is the optimal choice.

## Figures and Tables

**Table 1:** Device structure parameters used in simulations.

Parameters	Value
Source length (*L*_S_)	50 nm
Gate/source space (*L*_GS_)	50 nm
Left gate length (*L*_LG_)	100 nm
Drain length (*L*_D_)	50 nm
Right gate length (*L*_RG_)	100 nm
HfO_2_ length (*L*_HfO2_)	150 nm
P^+^-pocket length (*L*_p_)	100 nm
P^+^-pocket width (*W*_p_)	5 nm
InAlAs-block length (*L*_B_)	50 nm
InAlAs-block width (*W*_B_)	7 nm
SiO_2_ length (*L*_SiO2_)	100 nm
Gate/drain space (*L*_GD_)	50 nm
Bulk material width (W)	10 nm
Dielectric width (*W*_ox_)	2 nm
Left gate work-function	4.5 eV
Right gate work-function	5.3 eV

**Table 2:** Extracted parameters for three EHBTFETs.

Device	*I*_OFF_ (A/μm)	*I*_ON_ (A/μm)	*I*_ON_/*I*_OFF_	*V*_th_ (V)	*SS*_avg_ (mV/dec)	DIBL (mV/V)
V-EHBTFET	2.29 × 10^−16^	1.84 × 10^−5^	8.0 × 10^10^	0.26	30.2	N/A
VP-EHBTFET	8.37 × 10^−11^	1.04 × 10^−4^	1.2 × 10^6^	0.06	22.1	7.5
VPB-EHBTFET	1.83 × 10^−19^	1.04 × 10^−4^	5.7 × 10^14^	0.06	5.5	7.5

## Data Availability

The data presented in this study are available on request from the corresponding author.
